# Modeling of PEEK Crystallization Kinetics Under Transient Thermal Conditions

**DOI:** 10.3390/polym18070825

**Published:** 2026-03-27

**Authors:** Shahil Hamid, To Yu Troy Su, Soroush Azhdari, Abdullah Al Faysal, Patrick C. Lee, Sergii G. Kravchenko

**Affiliations:** 1 Department of Materials Engineering, The University of British Columbia, Vancouver, BC V6T 1Z4, Canada; shahil.hamid@ubc.ca (S.H.); sergey.kravchenko@ubc.ca (S.G.K.); 2 Department of Mechanical and Industrial Engineering, The University of Toronto, Toronto, ON M5S 3G8, Canadafaysal@mie.utoronto.ca (A.A.F.)

**Keywords:** crystallization kinetics modeling, transient thermal profile, additive manufacturing, fast scanning calorimetry

## Abstract

This study develops a kinetic model that captures poly-ether-ether-ketone (PEEK) crystallization over a temperature T window from glass transition ($T_{g}$) to melting ($T_{m}$) temperature, and across cooling rates from 5 to ~10^3^ °C/min. The framework is a parallel dual-Nakamura formulation whose isokinetic parameters {$k_{i}\left(T\right),n_{i},w_{i}\left(T\right)$} are obtained from a bi-level non-linear regression of isothermal crystallization tests conducted using a flash-differential scanning calorimeter (FSC). The weight $w_{i}\left(T\right)$ partitions the faster primary and slower secondary crystallization and is represented by a physics-based analytical function that captures its dome-shaped temperature dependence. A maximum isothermally achievable enthalpy function is introduced so that the model predicts enthalpy ΔH(t) natively under arbitrary thermal profiles. To extend this isothermal backbone to non-isothermal conditions, two explicit cooling-rate-dependent scalars are introduced, $\omega \left(\dot{T}\right)$ and $\chi \left(\dot{T}\right)$, which shift $w_{i}\left(T\right)$ and limit attainable crystallinity at high cooling rates respectively. Finally, a rate-dependent induction time relation is added to adjust the onset of crystallization. Calibrating these rate functions against non-isothermal experiments, while keeping the isokinetic parameters fixed, yields a single isothermal–non-isothermal model that predicts ΔH(t) under arbitrary T(t) profiles. Model performance is validated using an interrupted FSC experiment with a multi-segment cooling program that mimics a local transient thermal history of PEEK during additive manufacturing. The sample is cooled through successive constant-rate segments with intermittent quench–remelt cycles to probe the accumulated crystallinity along the path. Without additional fitting, the model predicts the measured enthalpy evolution with R^2^ ≈ 0.95. The framework thus provides a practical route for predicting polymer crystallinity under processing-relevant thermal histories.

## 1. Introduction

While crystallization kinetics modeling has received considerable attention for semi-crystalline polymers [1], most studies focus on simple conditions in which external variables, particularly temperature (T) and temperature rates ($\dot{T}$), are held constant [2,3,4,5,6]. Extending these models to arbitrary processing paths remains difficult for two main reasons. First, instrument limits, especially in conventional differential scanning calorimetry (DSC), restrict access to the full crystallization regime between glass transition temperature ($T_{g}$) and the melting temperature ($T_{m}$), such that measurements over $T_{g}<T<T_{m}$ are incomplete. In fast-crystallizing polymers, crystallization may initiate during the ramp to the target isotherm, compromising the isothermal test. Second, many semi-crystalline polymers, including poly-ether-ether-ketone (PEEK), exhibit dual crystallization mechanisms, so models calibrated under a single-mechanism assumption are unable to extend to arbitrary thermal profiles [2,7,8].

These limitations are important for PEEK, a semi-crystalline member of the poly(aryl-ether-ketone) (PAEK) family used in sectors such as oil and gas and aerospace for its thermal and chemical stability. The stiffness, strength, thermal stability, dimensional accuracy, and warpage of PEEK parts are governed by the degree of crystallinity (DoC) formed during cooling or annealing [1,2]. In additive manufacturing (AM) processes, such as fused-filament fabrication (FFF), steep thermal gradients and short dwell times can affect crystallization and part performance [3]. Process-path-aware models that can predict crystallization under AM-like thermal histories are therefore needed to support DoC control in 3D-printed polymer and polymer-based composite components.

Crystallization kinetic modeling begins with the Avrami [9] framework, which represents the evolution of crystallization under isothermal conditions as given by Equation (1):

(1) $$\alpha \left(t\right)\;=\;\left[1-\exp\left(-k(T)\;t^{\;n}\right)\right]$$

where k(T) is the temperature-dependent crystallization rate constant, n is the Avrami index describing nucleation and growth dimensionality, and $\alpha \left(t\right)$ denotes the relative DoC as a function of time. The Avrami model captures only the early stages of spherulitic growth because it assumes linear growth until impingement. However, this assumption fails for many polymers, including PEEK, as the transformation departs from linearity over time. This deviation implies constrained molecular mobility and the onset of a secondary process, variously interpreted as slow crystallization in inter-spherulitic amorphous regions or lamellar thickening [2,10,11,12]. This deviation is usually treated phenomenologically by writing $\alpha \left(t\right)$ as a function of the imposed thermal history T(t), with parameters obtained from calorimetric measurements. For PEEK, these measurements frequently show double-melting endotherms that are often associated with contributions from both primary and secondary crystallization [2,5,11], although other interpretations have been proposed [13]. To represent these contributions within a single kinetic framework, several authors have introduced explicit two-stage models.

Hillier [7,13] introduced a bi-stage modification that writes total conversion as a convolution of a primary Avrami process with a delayed secondary component, allowing sequential growth. Later, Velisaris and Seferis [2] offered a different perspective by modeling PEEK crystallization as two parallel nucleation and growth pathways. The faster primary process corresponds to three-dimensional spherulitic growth until impingement, while the slower secondary process involves one- or two-dimensional rod-like or epitaxial growth, separated by weight factors $w_{1}+w_{2}=1$ within each isothermal fit [2], as shown in Equation (2):

(2) $$\alpha \left(t\right)\;=\;\sum_{i\;=\;1}^{2}w_{i}\left[1-exp(-k_{i}(T)t^{n_{i}})\right]$$

where i = 1 and 2 represent primary and secondary crystallization respectively. Velisaris and Seferis [2] suggested the weights could vary with both (T) and ($\dot{T}$) principles. However, as the available instrumentation could not fully acquire isothermal data over the range $T_{g}<T<T_{m}$, the authors were constrained to treat $w_{i}$ as constants with respect to temperature and to allow variation only with cooling rate [2]. Later, Seo et al. [6] reformulated the parallel Avrami model (Equation (2)) by replacing $w_{1}$ and $w_{2}$ with probabilities tied to the evolving crystallinity, so that the kinetics are governed by the primary mechanism at low crystallinity and then shift beyond a predetermined transition crystallinity to a slower secondary mechanism. While this model provides an alternative description of isothermal crystallization kinetics, Driezenn and Herrmann [14] demonstrated in their work that extending it to non-isothermal conditions requires further conceptualizations.

Other kinetic descriptions that do not rely on the traditional Avrami framework have also been proposed [10,15,16]. Veyrat Cruz-Guzman et al. [10] used a rate equation written with a fractional Caputo derivative of order between zero and one, which introduces a memory of the prior thermal history; within this history-dependent law, the same kinetics describe both the initial primary crystallization and the long-time secondary crystallization, without an explicit second term. A different ‘model-free approach’ has also been proposed by Vyazovkin and Wight [16] and later adopted by Gordnian [15] to describe PEEK crystallization kinetics. In a comparative study, Kelly and Jenkins [3] fitted eight different isothermal models, including single Avrami [9] (Equation (1)), parallel Velisaris–Seferis (Equation (2)) [2] and Hillier [7] to poly(3-hydroxybutyrate-co-3-hydroxyvalerate) crystallization. Their analysis suggested that the parallel Velisaris–Seferis equation produced better fits [3].

Non-isothermal modeling is usually treated as an infinite sum of isothermal segments [2,17,18,19,20], although other non-integral-based approaches also exist [15,21]. For example, under constant cooling, Ozawa [21] re-expressed the Avrami equation enabling estimation of n at different $\dot{T}$. However, Ozawa’s method requires evaluating α(t) at the same temperature for several $\dot{T}$ values and cannot be extended to arbitrary thermal profiles; it also omits secondary mechanisms [19].

The integral Avrami approach implemented by Nakamura et al. [20] is expressed by Equation (3):

(3) $$\alpha \left(t\right)\;=\;1-exp\left({-\left[\int_{0}^{t}{k(T(\tau ))}^{\frac{1}{n}}\;d\tau \right]}^{n}\right)$$

which reduces to Avrami (Equation (1)) under isothermal conditions. Building on Velisaris and Sefaris’ [2] superposition idea, Pérez-Martín et al. [4] implemented a parallel integral dual-Nakamura formulation for modeling non-isothermal crystallization of poly-ether-ketone-ketone (PEKK) and fiber-reinforced PEKK composites. For the isothermal holds, the DSC data were first fitted with the Velisaris–Seferis model (Equation (2)): $n_{1}\;and\;n_{2}$ were taken as global values for each material, $w_{i}$ at each crystallization temperature were assigned using the ratios of the measured low-temperature (LT) and high-temperature (HT) endotherms, and the corresponding $k_{1}$ and $k_{2}$ were obtained by non-linear least-squares fitting. These temperature-dependent kinetic parameters were then inserted into the dual-Nakamura formulation to predict crystallization during continuous cooling, with $n_{1}\;and\;n_{2}$ held fixed and $w_{1}$ allowed to vary empirically with $\dot{T}$ to match the non-isothermal DSC $\alpha \left(t\right)$ evolution.

Bessard et al. [5], and later Driezen and Herrmann [14], adopted a parallel differential dual-Nakamura (numerically equivalent to the integral version [19]) model with separate $\left(n_{i},k_{i}\left(T\right)\right)$ for each crystallization mechanism and a weighting structure to partition the contributions across the thermal history. In Bessard et al.’s [5] work, the isothermal DSC holds were first fitted by identifying {$w_{1},k_{1},k_{2},n_{1},n_{2}$} at each crystallization temperature by numerical optimization. The $n_{1}\;and\;n_{2}$ parameters were then treated as temperature-independent. For constant-rate non-isothermal cooling, the cooling path was discretized in time and the differential dual-Nakamura model was used with $n_{1}\;and\;n_{2}$ fixed to their isothermal values, while $w_{1},k_{1},\;and\;k_{2}$ parameters were re-adjusted for each cooling rate so that the predicted $\alpha \left(t\right)$ matched the DSC curves. Driezen and Herrmann [14] likewise employed a differential dual-Nakamura formulation, determining {$k_{1},k_{2},n_{1},n_{2}$} from isothermal flash-DSC (FSC) data across the crystallization window. During non-isothermal prediction, primary and secondary contributions were combined using time-dependent weight factors based on the instantaneous α, analogous to Seo’s probability-based scheme.

Collectively, these dual-Nakamura-based studies improved the prediction fidelity of constant-rate, non-isothermal crystallization. However, in most cases, the kinetic parameters were calibrated separately on isothermal and non-isothermal datasets [2,4,5], so the link between isothermal and non-isothermal behavior is indirect. Parameter identification was also typically restricted to relatively narrow, high-temperature windows near $T_{m}$ and to slow or moderate cooling rates, which makes extrapolation toward the rapid, transient cooling histories relevant to polymer processing uncertain [2,4,5,10,15,22]. Moreover, explicit handling of secondary evolution [2,5,6,14] and induction effects [5,15,23] varies across studies. To the best of our knowledge, model performance is usually assessed against simple isothermal curves and a limited set of constant-rate cooling experiments. It therefore remains unclear how reliably these parameter sets describe crystallization under complex non-linear transient thermal profiles.

The instrumentation used in thermal analysis constrains the crystallization behavior that can be modeled and validated. Much of the earlier literature presented thus far is anchored in DSC at rates ≤ 60 °C/min and limited to high-temperature isothermal windows near $T_{m}$ [2,4,5,10,15,22]. The advent of FSC extended access across the full $T_{g}<T<T_{m}$ window by allowing cooling rates as high as ~10^7^ °C/s. Both Tardif et al. [13] and Seo et al. [6] used FSC to implement an isothermal model but did not demonstrate non-isothermal or transient-rate verification. Driezen and Herrmann [14] and Comelli et al. [24] utilized non-isothermal rates up to ~10^3^ °C/min and collected full $T_{g}<T<T_{m}$ isothermal data, yet explicit T and $\dot{T}$-dependent weight-factor evaluation and validation under complex transient cooling profiles were not reported.

The present treatise assembles a parallel dual-Nakamura framework for PEEK using isokinetic parameters {$w_{i}(T),k_{i}(T),n_{i}$} calibrated over $T_{g}<T<T_{m}$ using FSC isothermal crystallization tests. The isothermal data are fitted with the Velisaris–Seferis model (Equation (2)) using a physics-based constrained optimization to extract the isokinetic parameters. Smooth and continuous analytical temperature functions are proposed for each parameter so that their variation with T is physically consistent across the temperature range. These isokinetic parameter functions are then inserted, without modification, into a dual-Nakamura formulation with additional calibration against constant-rate dynamic tests by means of two proposed $\dot{T}$-dependent scalars. Direct deployment of the isothermal model alone within the dual-Nakamura formulation leads to systematic overprediction of crystallization at high cooling rates, indicating that T dependence alone is insufficient to capture non-isothermal kinetics. To address this, two explicit $\dot{T}$-dependent scalers, $\omega \left(\dot{T}\right)$ and $\chi \left(\dot{T}\right)$, are introduced. The $\omega \left(\dot{T}\right)$ parameter shifts $w_{i}(T)$ to redistribute the relative contributions of the primary and secondary mechanisms, while $\chi \left(\dot{T}\right)$ acts as a constrained global scalar that suppresses the absolute DoC under rapid cooling. To remove ambiguity associated with relative crystallinity under transient profiles, the model incorporates T-dependent maximum achievable enthalpy under isothermal conditions $\Delta H_{max}^{\mathrm{iso}}\left(T\right)$, following Driezen and Herrmann [14], so that the framework natively predicts absolute enthalpy $\Delta H\left(t\right)$ for arbitrary T(t). A new induction time logic, tailored for transient rates and based on Godovsky’s power law [25], is added to delay the onset of crystallization as a function of cooling rate. Collectively, these elements define a unified framework for predicting DoC evolution under complex transient AM-relevant thermal histories.

To validate the predictive capability of the developed crystallization kinetics model, an interrupted FSC test following a complex cooling profile is proposed. The sample is cooled in the FSC along a prescribed multi-segment profile that mimics the non-linear local cooling profile in AM. The sample is quenched and reheated to melt between segments, allowing crystallization to be probed at several interruption points along the path. The fully calibrated model is deployed directly, without any further parameter adjustment, to predict the evolving $\Delta H\left(t\right)$. The results of the validation experiment demonstrate that the developed crystallization kinetics model connects isothermal and non-isothermal behavior correctly, enabling forward prediction of crystallinity under AM-relevant processing thermal histories. The proposed model potentially supports the design of PEEK components with predictable properties.

## 2. Materials and Methods

The PEEK utilized in this work is the CF10 LS1 3D Printing grade PEEK filament, supplied by Solvay Additive Manufacturing (Brussels, Belgium) and used without modification. Prior to thermal analysis, the PEEK is dried at 120 °C for 8 h in an oven under vacuum to remove moisture. Calorimetric testing is conducted under an inert atmosphere of continually flowing laboratory grade N_2_, purchased from Air Liquide (Paris, France) and used as received.

### 2.1. FSC Sample Preparation and Mass Determination

The FSC used in this work is the Flash DSC 2+ (equipped with an intracooler and optical microscope) and the FSC chips are the UFS-2 chips, all purchased from Mettler Toledo (Columbus, OH, USA). Prior to use, the chips are first conditioned and calibrated, both of which are standardized processes that involve executing pre-programmed methods in the software, StarE V18.0, also provided by Mettler Toledo. Conditioning and calibration are intended to correct for errors in the chip’s measurement accuracy, not to correct for sample preparation errors. As such, no calibration materials are used. As defined by Mettler Toledo, a well-conditioned chip is one that exhibits less than 130 μW drift in heat flow reading between 50 °C and 400 °C. The calibration process defines a set of parameters to relate measured temperature with actual temperature, and as such, has no acceptance criteria. A conditioned and calibrated chip is placed into the FSC prior to the start of sample preparation.

Samples are prepared by shaving small pieces of PEEK off the filament and onto a cleaned glass slide with a scalpel under an optical microscope. The shaving is halved using a scalpel until it fits the sensor area of the FSC chip. There is variability expected in the mass of each prepared sample, which is calculated after sample preparation for the purposes of heat flow normalization. Using a single synthetic and cleaned hair, the PEEK sample is transferred from the glass slide and positioned onto the center of the sensor area of the chip. The FSC sample chamber is closed and a steady flow of 80 mL/min N_2_ is introduced to the sample chamber. The intracooler is turned on, which lowers the chamber temperature to −60 °C.

Once stabilized at −60 °C, the sample is melted onto the sensor chip by heating the sample to 400 °C at a rate of 10 °C/min holding isothermally for 1 s, then cooling down slowly to −60 °C at a rate of −10 °C/min. To ensure that the sample is fully melted, three heat–cool cycles are executed in rapid succession at realistic operating heating and cooling rates of 4000 °C/s and −2000 °C/s, respectively. Lastly, the machine is heated back to room temperature, and the centering of the sample is verified by optical microscopy. A nominal sample is well-adhered to the chip membrane within the sensor area. This procedure is repeated for each FSC chip prepared. FSC chips and their samples are retired after they have cumulatively spent 20 min at temperatures in excess of 350 °C, because preliminary tests showed that the PEEK undergoes significant thermal degradation under these conditions, thereby reducing subsequent enthalpy measurements. A decline in relative enthalpy by 20% is considered to be significant and warrants the sample in question’s retirement.

The thermal analyses performed on the data extracted from FSC samples require a measurement of its mass. Considering its small size (80–140 nanograms), the mass of FSC samples is calculated as a ratio of melting enthalpies of two samples, one of a known mass, and one of the FSC sample of an unknown mass. To obtain the melting enthalpy of a sample of a known mass, a DSC sample is prepared in accordance with ASTM E967-18 [26]. The DSC used in this work is the Netzsch DSC214 Polyma (Selb, Germany) equipped with a liquid nitrogen cooling accessory, allowing it to heat and cool at rates between 0.0001 and 500 °C/min. During DSC operation, a steady flow of 40 mL/min N_2_ gas is introduced over the sample to create an inert atmosphere. A PEEK sample of 5–10 mg is measured using a laboratory scale ($m_{DSC}$) and sealed inside of a standard aluminum hermetic pan provided by Netzsch. Both the DSC sample of known mass and the FSC sample of unknown mass are subjected to identical cooling profiles of −40 °C/min from 400 °C to 25 °C to align their thermal histories. The subsequent melting enthalpy upon reheating is measured, yielding specific enthalpy (${∆H}_{m,\;DSC}$) from the DSC sample and non-mass normalized enthalpy (${∆H}_{m,\;FSC}$) from the FSC sample. The ratio of these two quantities yields the mass of the FSC sample ($m_{FSC}$), as per Equation (4), correlated by the ratio of heating rates.

(4) $$m_{FSC}=\frac{{∆H}_{m,\;FSC}}{{∆H}_{m,\;DSC}}\cdot m_{DSC}$$

### 2.2. FSC Experiments

In this work, the FSC is used to conduct all of the isothermal and non-isothermal tests other than the slower 5 and 10 °C/min rate tests. In traditional DSC, analysis of the isothermal and/or linear cooling segments yields relevant time-series heat flow data about the crystallization kinetics and evolution of crystallization. Due to the smaller FSC sample mass and subsequently small heat flow signal, especially during isotherms, thermal analysis in these segments is impossible. To work around this, researchers have adopted what is known as a stepwise method [13,24,27,28], wherein upon cooling or isotherm, the sample is quenched to instantaneously “freeze” the sample in its state, then analyzed upon subsequent re-heating. By repeating this process and gradually increasing the duration of isotherm/non-isothermal cooling at each step, the time-series evolution of crystallization is revealed.

**Figure 1:**
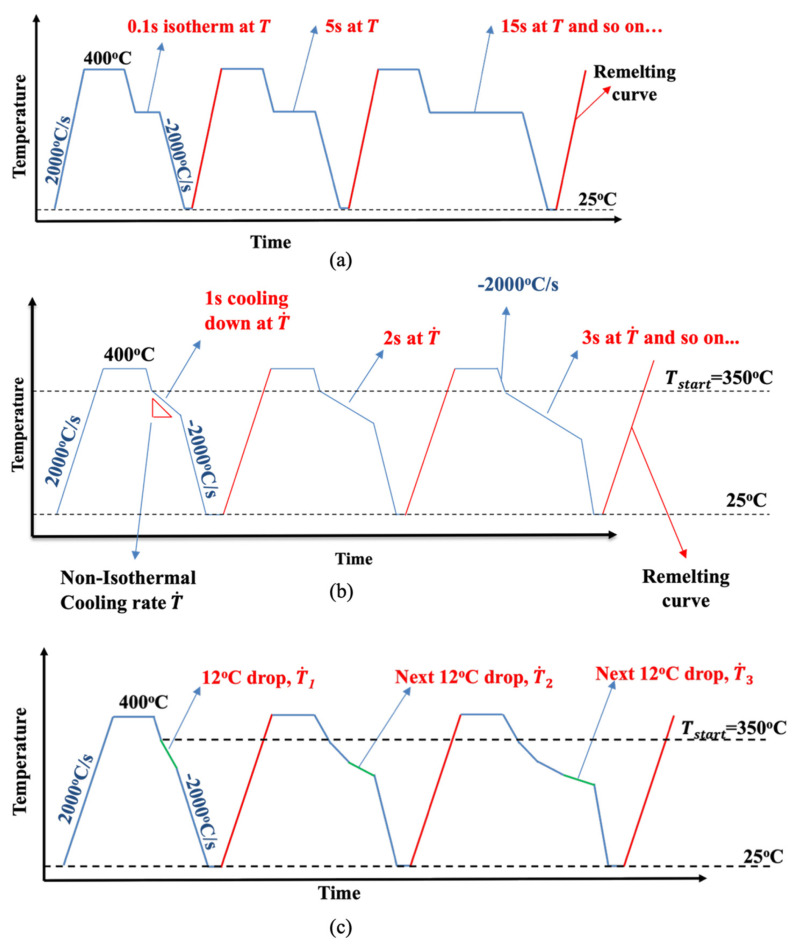
FSC stepwise thermal profile for testing (**a**) isothermal, (**b**) non-isothermal crystallization kinetics and (**c**) verification thermal profile. Red lines indicate heating cycles where effect of crystallization induced by previous cooling profile is measured. Green lines indicate the added segments between cycles of the stepwise profile.

To implement the stepwise method for isothermal tests, samples are first cyclically melted and crystallized to erase thermal history. Beginning at 400 °C, samples are quenched to desired isothermal temperatures and held for a specified isothermal duration before being quenched again to −60 °C. At each temperature extreme, samples are held isothermally for 0.1 s to mitigate effects of thermal lag. Upon subsequent reheating to 400 °C, the melt enthalpy is measured and assumed to be wholly consistent of energy absorption due to the melting of crystals formed during the preceding isotherm step. The next heat–quench–isotherm–quench–heat cycle will see an increase in isotherm duration. For this study, isotherm temperatures spanning 155–310 °C are tested for durations of 0.1–1440 s on the FSC. See Figure 1a for the stepwise isothermal thermal profile.

Implementing the stepwise method for non-isothermal tests is similar, except samples are first quenched from 400 °C to 350 °C (avoiding the window of thermal degradation) followed immediately by constant cooling at the $\dot{T}$ of interest (see Figure 1b). Given Poel et al.’s [29] observation of only a 0.8 °C lag at 1000 °C/s in the FSC, and the fact that crystallization onsets for the slowest non-isothermal rate tested (120 °C/min) start below 280 °C (and even later for faster ramps), this pre-segment does not measurably bias the onset or growth. In these tests, the cooling duration is represented by an ending temperature, whereby a lower ending temperature implies a longer duration of cooling. For this study, cooling rates of 120–3500 °C/min and start and end temperatures of 280–180 °C respectively were evaluated on the FSC.

To demonstrate the efficacy of the model developed in this study, a non-linear cooling profile based on experimental measurements of material temperature during AM between 350 and 175 °C was developed. This temperature profile was then approximated by 15 linear cooling segments of rates ranging from 1000 to 0 °C/min. In accordance with the stepwise method described above, the sample is heated to 400 °C to fully melt all crystals, then quenched to 350 °C before executing the approximated non-linear profile. In each cycle of the stepwise method, an additional segment is added until the entire approximated non-linear profile is performed in the final cycle (see Figure 1c). Following each cycle, the contribution to subsequent melt enthalpy from the added segment is analyzed upon reheating and a time-evolution of crystallization is developed for comparison with the model output. The segmented cooling rates are presented in Table 1.

### 2.3. DSC Experiments

In instances where analysis of slow cooling rates would result in the FSC sample exceeding the acceptable limit for thermal degradation (i.e., sample spends more than 20 min heated above 350 °C), the DSC is used to supplement. When using the DSC, the stepwise method is unnecessary, as analysis can be adequately performed on the cooling cycles due to the larger sample mass. The same Netzsch DSC214 Polyma setup mentioned in Section 2.1 is used for conducting the 5 and 10 °C/min non-isothermal tests.

### 2.4. Enthalpy Analysis and α(t) Calculation

Once calorimetric tests are conducted, time–temperature–heat flow data is exported from either StarE (FSC) or Proteus (DSC) for processing in MATLAB R2024a. Prior to processing, the data is smoothed using a Savitzky–Golay filter [30] (sgolayfilt function in MATLAB Signal Processing Toolbox). If the dataset originates from an FSC sample, the heat flow is also normalized (specific heat flow) against the mass, as determined by the method outlined above. The calculation of specific enthalpy is often cited as a simple numerical integration of heat flow over the relevant domain of time for each enthalpic event [31]. This simplification masks the subjectivity inherent to the construction of a baseline heat flow to which one numerically integrates the heat flow signal.

In this work, we explicitly account for the fact that the measured heat flow signal across a melting or crystallization event contains contributions from both latent heat and changes in baseline heat capacity [32]. To separate these contributions, we construct a sigmoidal baseline as a weighted average of two linear baselines, each tangent to the experimental signal immediately before and after the enthalpic event, following the general procedure described by Höhne et al. [32]. This sigmoidal baseline is implemented numerically in MATLAB and applied to all enthalpy calculations to ensure consistency during analysis and minimization of user subjectivity.

α(t) is calculated as the ratio of the melting enthalpy measured upon reheating at t to the melting enthalpy obtained at the final remelting endotherm. The enthalpy measured at this final remelting step is termed the maximum achievable enthalpy ${\Delta H}_{max}$ detailed in Equation (5).

(5) $$\alpha \left(t\right)=\frac{∆H(t)}{{\Delta H}_{max}}$$

## 3. Isothermal Crystallization Kinetics Modeling

The objective of the isothermal analysis is to identify the isokinetic parameters that (i) reproduce the measured α(t) at each T, (ii) connect cleanly to a temperature-dependent law for later interpolation, and (iii) can be projected to non-isothermal histories in the final unified model.

### 3.1. Translating Calorimetric Measurements into Model Parameters

**Figure 2:**
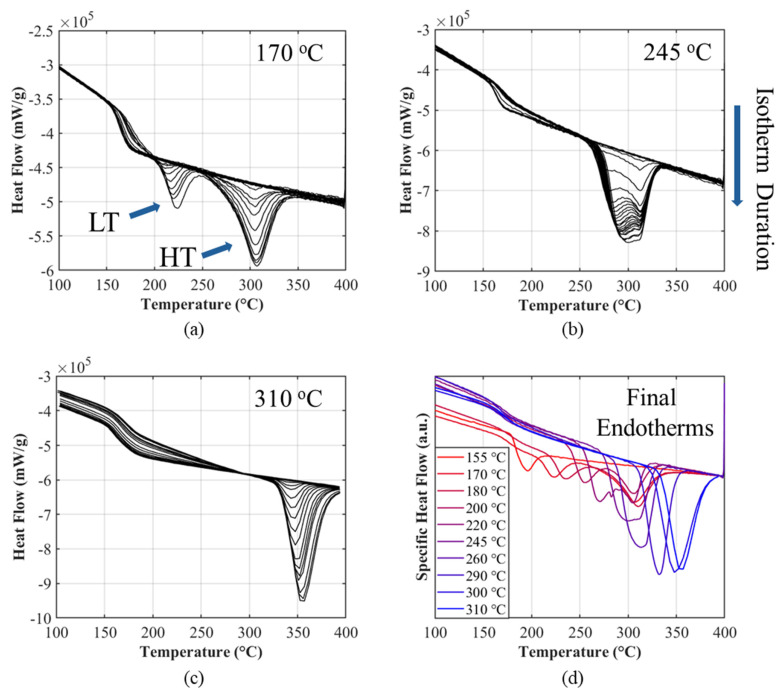
Stepwise isothermal melting endotherms obtained from the FSC: (**a**) 170, (**b**) 245, (**c**) 310 °C and (**d**) the final melting endotherms of each test.

Ten isothermal FSC experiments were performed across the full crystallization window of PEEK (155–310 °C) using the stepwise protocol described in Section 2. For each isothermal crystallization temperature, the final remelting curve after the longest hold was integrated to define $∆H_{max}^{\mathrm{iso}}\left(T\right)$. The resulting $∆H_{max}^{\mathrm{iso}}\left(T\right)$ profile forms a right-skewed bell-shape with a single maximum near ≈ 290 °C, consistent with reported values from Driezen and Herrmann’s [14] FSC study. At isothermal crystallization temperatures > 245 °C, a single dominant peak appears and remains comparatively narrow. As T is reduced, the peak broadens and splits, revealing an LT and an HT endotherm whose separation grows as T approaches $T_{g}$. The HT peak sharpens and translates systematically with the isothermal setpoint; the LT endotherm occurs reproducibly ≈ 30–40 °C above the isothermal T; and both peaks evolve consistently with extended hold time. These trends align with Bassett’s [11] lamellar thickening interpretation, in which lamellae thicken during extended hold times and more perfect (stable) crystals form at higher crystallization temperatures. Concomitantly, the $∆C_{p}$ at $T_{g}$ becomes less pronounced with increasing hold, an effect ascribed to the reduction in mobile amorphous fraction as crystallinity develops, consistent with Tardif et al.’s FSC results [13]. Representative isothermal sets at 170 °C, 245 °C, and 310 °C are plotted in Figure 2a–c to highlight the characteristic signatures across the window, while Figure 2d compiles the final endotherms of all the isotherms tested to visualize the dual peak behavior.

The α(t)∈ [0,1] (Equation (5)) evolution from each isothermal calorimetric measurement is fitted to the parallel Avrami equation (Equation (2)) for parametrization. The weight factors are allowed to vary against temperature where $w_{1}\left(T\right)+w_{2}\left(T\right)=1$, $0<w_{i}\left(T\right)<1$, for $T\in [T_{g},T_{m}^{0}]$. In what follows, the exponents {$n_{1},n_{2}$} are treated as global (temperature-invariant) parameters, whereas $\left\{k_{1}\left(T\right),k_{2}\left(T\right),w_{1}\left(T\right)\right\}$ are local to each isothermal condition. The initial short-hold interruptions yield no discernible melting endotherm upon the reheating segments. The earliest points of each α(t,T) begin at the first nonzero enthalpy; this naturally excludes the induction period (no growth detected), so that fits are anchored to the onset of measurable crystal content rather than to an arbitrary “zero-time” chosen on the instrument trace [12].

**Figure A2:**
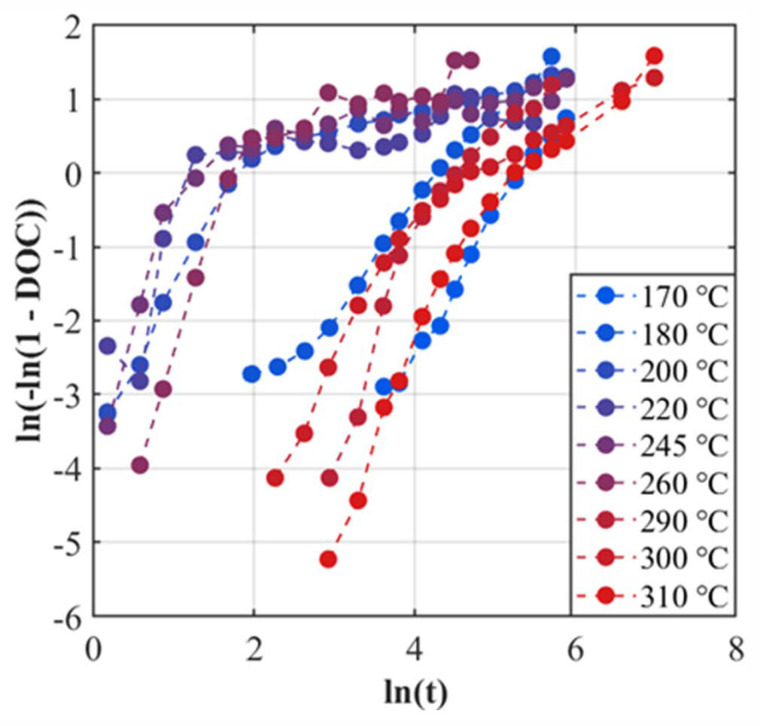
Bi-segmented linearized Avrami fits of the isothermal tests.

Direct bi-linear fits on the double-log Avrami plot (see Figure A2) indeed reveal two distinct regimes (primary and secondary) but when used to estimate $k_{1}\left(T\right)$, $k_{2}\left(T\right)$, $n_{1}$, and $n_{2}$ independently at each T, they routinely generate non-physical secondary exponents values of $n_{2}<1$, an issue also documented by Driezen and Herrmann [14] and Pérez-Martín et al. [4]. To remove this inconsistency while preserving the observed two-regime behavior, parameter estimation was performed using a bi-level constrained regression [33].

For each trial pair, $n_{1}$ and $n_{2}$, the local triplet $\theta_{i,BL}=\{k_{1}\left(T_{i}\right),k_{2}\left(T_{i}\right),w_{1}\left(T_{i}\right)\}$ is obtained by minimizing the per-isotherm error subject to the following constraints: $0<w_{1}<1$, ${3\leq n}_{1}\leq 4$, ${1<n}_{2}\leq 2$ and $k_{i}\left(T\right)>0$. This ensures $n_{1}$ and $n_{2}$ represent 3D spherulitic and 1 or 2D rod-like or epitaxial growth respectively [34]. The outer (global) search is performed with particle swarm optimization (PSO) which is well-suited to low-dimensional problems with potentially noisy, non-smooth objectives, as is the case here because the objective value is the sum of results from stochastic inner optimizations [35]. It is also relatively fast to converge to good solutions in such settings compared to a genetic algorithm (GA) (used in the inner level problem) [36,37]; the inner problems are solved with GA. The implementation was carried out in MATLAB R2024a using the Global Optimization Toolbox. Numerical implementation of this bi-level non-linear regression is mentioned in the Appendix A.

**Figure 3:**
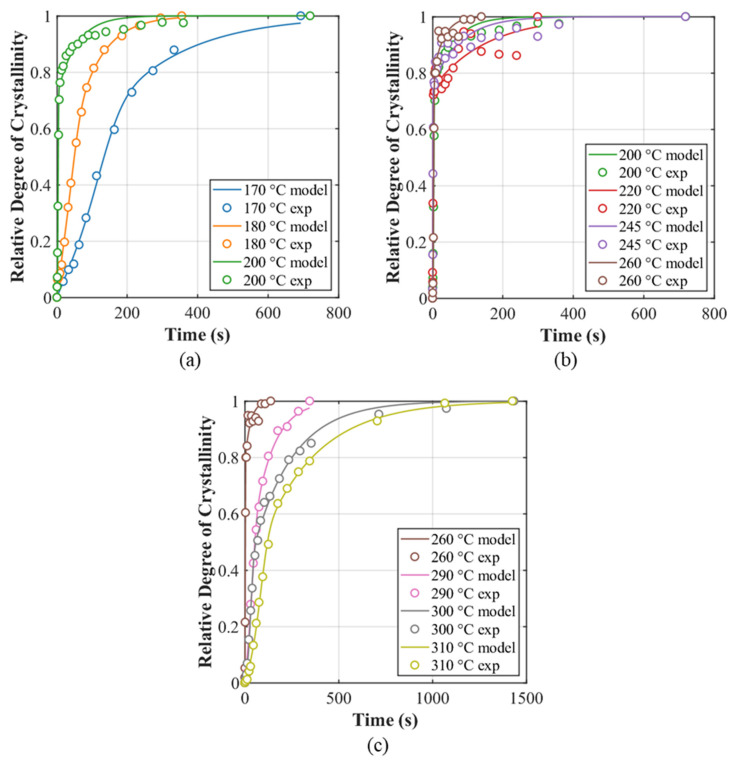
Relative crystallinity plots of experimental and model prediction of (**a**) 170, 180, 200 °C, (**b**) 200, 220, 245, 260 °C and (**c**) 260, 290, 300, 310 °C isothermal tests.

The final outer solution returned the global exponents $n_{1}$ = 3.000 and $n_{2}$ = 1.001. The corresponding per-temperature results for ${\{\theta }_{i,BL}=\{k_{1}\left(T_{i}\right),k_{2}\left(T_{i}\right),w_{1}\left(T_{i}\right)\}$ are reported in Table 2, and representative $\alpha \left(t\right)$ overlays (model vs. experiment) are provided in Figure 3a–c.

### 3.2. Analytical Fitting of $k_{i}(T)$, $w_{1}(T)$ and $\Delta H_{max}^{\mathrm{iso}}(T)$

Having identified {$k_{1}\left(T\right),k_{2}\left(T\right),w_{1}\left(T\right)$} and $\Delta H_{max}^{\mathrm{iso}}(T)$ at discrete isotherms, we now provide closed-form temperature laws so the isothermal results can be interpolated across $T_{g}<T<T_{m}$ and carried into the non-isothermal model. Details of the parameter optimization procedures associated with each interpolation function presented in the following subsections are provided in the Appendix A.

#### 3.2.1. Temperature Laws for the Isokinetic Rate Constants $k_{i}\left(T\right)$

In the parallel Avrami model, $k_{1}$ and $k_{2}$ (primary and secondary respectively) are treated as effective volumetric crystallization rates that combine nucleation and lamellar growth contributions for the primary and secondary mechanisms, respectively. Their bell-shaped temperature dependence is described using the standard Hoffman–Lauritzen expression [4,38] given in Equation (6):

(6) $$k_{i}\left(T\right)\;=\;K_{0,i}\cdot \exp\left(-\frac{d_{i}U_{i}^{*}}{R\left[T-T_{\infty ,i}\right]}\right)\cdot \exp\left(-\frac{d_{i}K_{g,i}}{T\left[T_{m,i}^{0}-T\right]{f\left(T\right)}_{i}}\right)$$

(7) $${f\left(T\right)}_{i}=\frac{2T}{T_{m,i}^{0}+T}$$

where $∆T=T_{m}^{0}-T$ is the supercooling; $T_{m}^{0}$ is the equilibrium melting temperature; $T_{\infty }\approx T_{g}-30$K is the temperature where segmental mobility ceases; $U^{*}$ is the activation energy for segmental transport; R=8.314J/(mol·K) is the universal gas constant; $K_{g}$ is the nucleation constant (theoretically related to fold surface free energy and lamellar thickness); and f is the correction factor for temperature dependence of the surface free energy. Pre-exponential $K_{0,i}$ lumps $N_{i}\left(T\right)$. In our implementation, we fix $d_{1}=3\;and\;d_{2}\;=\;2$ representing three- and two-dimensional growth respectively, as suggested by Pérez-Martín et al. [4]. All temperatures are in kelvin. Therefore, the unknowns for each $k_{i}\left(T\right)$ are collected in the decision vector $\theta_{i,k}=\left\{K_{0,i},K_{g,i},U_{i}^{*},T_{m,i}^{0},T_{\infty ,i}\right\}.$

To restrict the search to physically reasonable values, the parameters were initially bounded to finite intervals using the following box constraints: $K_{0,i}\in \left[10^{2},10^{6}\right]$ s^(−2,−3)^, $K_{g,i}\in \left[10^{4},10^{8}\right]$ K^2^, $U_{i}^{*}\in \left[10^{3},10^{4}\right]$ J/mol, $T_{m,i}^{0}\in \left[600,720\right]$ K and, $T_{\infty ,i}\in \left[330,450\right]$ K. However, as $k_{1}$ exhibits a sharper, narrower temperature peak, the $K_{0,1}$ bounds were later increased to $\left[10^{12},10^{20}\right]$ s^−3^ to allow for numerical convergence.

**Figure 4:**
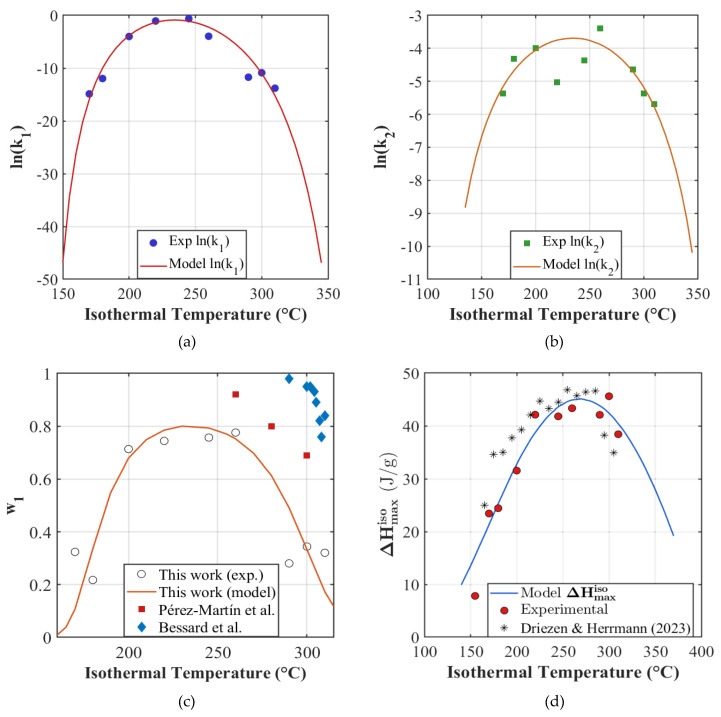
(**a**) Crystallization rate constants $ln(k_{1})$ and (**b**) $ln(k_{2})$ fitted with Hoffman–Lauritzen equation, (**c**) $w_{1}$ data points plotted against branching fraction model and other authors [4,5] and (**d**) $\Delta H_{max}^{\mathrm{iso}}$ data points plotted against the Michaelis–Menton-type function and other author [14].

The optimized Hoffman–Lauritzen parameters obtained from the fit are listed in Table 3. Our fitted parameters indicate that primary crystallization has larger effective barriers ($U_{1}^{*}>U_{2}^{*}$ and $K_{g,1}>K_{g,2}$) and a reduced crystallizable temperature interval ($T_{\infty ,2}\;\&\;T_{m,2}^{0}\;>T_{\infty ,1}\;\&\;T_{m,1}^{0}$). The large $K_{0,1}$ and smaller $K_{0,2}$ are consistent with higher effective primary nucleation/activity and weaker secondary nucleation, respectively, and are not mere artifacts of fitting [39]. As a result, the $k_{1}\left(T\right)$ curve is narrower and more sensitive both to the loss of mobility near $T_{\infty ,1}$ and to the collapse of supercooling near $T_{m,1}^{0}$. In contrast, $k_{2}\left(T\right)$ has smaller barriers and thus a broader temperature dependence, allowing it to persist more effectively at the edges of the crystallization window (see Figure 4a,b).

#### 3.2.2. Weighting as a Branching Fraction

The constrained bi-level extracted $w_{1}\left(T\right)$ from Section 3.1 demonstrates a dome-shaped trend rising to a maximum in the intermediate window and tapering towards zero as T approaches either $T_{g}$ or $T_{m}$ (see Figure 4c). These trends are consistent with our interpretation that primary crystallization is a space-filling process that requires both sufficient segmental mobility and a finite driving force; it therefore dominates at intermediate temperatures where both are favorable but is suppressed near $T_{g}$ (mobility loss) and near $T_{m}$ (vanishing undercooling for nucleation). In contrast, the secondary mechanism, which we associate with smaller effective barriers, can remain active toward the edges of the crystallization window [39].

To encode this behavior in a compact analytic form, we adopt the standard branching fraction representation inspired by parallel chemical pathways: the yield of a channel is its propensity divided by the sum of propensities [40,41]. Let

(8) $$w_{1}\left(T\right)=\frac{{\left[k_{1}\left(T\right)\right]}^{p_{1}}}{{\left[k_{1}\left(T\right)\right]}^{p_{1}}+\lambda {\left[k_{2}\left(T\right)\right]}^{p_{2}}}$$

Here, $k_{i}\left(T\right)$ is calculated with Equation (6) with its optimized parameters, and $p_{i}>0$ are adjustable exponents that allow for curvature tuning. With λ>0, this expression ensures that $0<w_{1}\left(T\right)<1$ for all T, and provides the logistic-like curvature needed to capture the shoulder inflections and the maxima. The asymptotic behavior of this expression can be understood from the Hoffman–Lauritzen form of the rate constants. Near $T_{g}$, $k_{i}\left(T\right)\sim \exp\left[-U_{i}^{*}/R\left(T-T_{\infty }\right)\right]$, while near $T_{m}^{0}$, $k_{i}\left(T\right)\sim \exp\left[-K_{g,i}/\left(T∆Tf\right)\right]$. The limiting value of the ratio depends on the relative magnitudes of the barrier terms:

(9) $$\underset{T\to {T_{g}}^{+}}{\lim}w_{1}\left(T\right)\;=\;\left\{\begin{array}{c} 0,\;p_{1}U_{1}^{*}>p_{2}U_{2}^{*}\\ 1,\;\;\;\;\;p_{1}U_{1}^{*}<p_{2}U_{2}^{*}\\ const,\;p_{1}U_{1}^{*}=p_{2}U_{2}^{*} \end{array}\right.\;,\underset{T\to T_{m}^{0-}}{\lim}w_{1}\left(T\right)\;=\;\left\{\begin{array}{c} 0,\;p_{1}K_{g,1}>p_{2}K_{g,2}\\ 1,\;\;\;\;\;p_{1}K_{g,1}<p_{2}K_{g,2}\\ const,p_{1}K_{g,1}=p_{2}K_{g,2} \end{array}\right.$$

We consider $w_{1}\left(T\right)$ function on the open interval $\left(T_{g},T_{m}^{0}\right)$, so $w_{1}\left(T\right)\in \left(0,1\right)$ for all admissible T. This is because we consider that, physically, both primary and secondary processes are expected to contribute at all temperatures, but their relative weights vary strongly across the crystallization window. The one-sided limits $\underset{T\to {T_{g}}^{+}}{\lim}w_{1}\left(T\right)=0$ and $\underset{T\to T_{m}^{0-}}{\lim}w_{1}\left(T\right)=0$ imply that $w_{1}\left(T\right)$ approaches zero at the boundaries, but is never exactly zero at any finite $T\in \left(T_{g},T_{m}^{0}\right)$. Thus, imposing the inequalities $p_{1}U_{1}^{*}>p_{2}U_{2}^{*}$ and $p_{1}K_{g,1}>p_{2}K_{g,2}$ ensures that $w_{1}\left(T\right)$ vanishes at both ends of the temperature window. The optimized fitting parameters $p_{1}$, $p_{2}$ and λ are listed in Table 4 and Figure 4c displays the model fit. Figure 4c also compares $w_{1}\left(T\right)$ extracted from the present work to the isothermal DSC analyses of Pérez-Martín et al. [4] and Bessard et al. [5]. These prior studies were restricted to only the high-temperature part of the window and consequently do not reproduce the full dome-shaped dependence as observed here.

#### 3.2.3. Maximum Isothermally Achievable Enthalpy $\Delta H_{max}^{\mathrm{iso}}\left(T\right)$

The $\Delta H_{max}^{\mathrm{iso}}\left(T\right)$ term is defined as the portion of a thermodynamic ceiling that is kinetically accessible at the chosen isotherm during the FSC protocol. It is equivalent to the denominator of Equation (5) but only for isothermal tests.

Under an arbitrary T(t) profile, the relative crystallinity evolution does not provide any relevant information regarding the absolute crystalline state of the sample [14]. This limitation is addressed by expressing the final absolute enthalpy as the sum over infinitesimal segments of the product of $\Delta H_{max}^{\mathrm{iso}}\left(T\right)$ and α(t) as discussed later in Section 4.2. The $\Delta H_{max}^{\mathrm{iso}}\left(T\right)$ is expressed as Equation (10):

(10) $$\Delta H_{max}^{\mathrm{iso}}\left(T\right)\;=\;∆H_{eq}\left(T\right)\eta \left(T\right)$$

where $\eta \left(T\right)\in \left[0,1\right]$ is defined as the kinetic factor and $∆H_{eq}\left(T\right)$ reflects the equilibrium crystallization enthalpy that could be realized if the system reached its thermodynamic limit at T. $∆H_{eq}\left(T\right)$ reflects the equilibrium degree of crystallinity $\alpha_{eq}\left(T\right)$, fundamentally determined by the free-energy gradient (∆G) and lamellar thickness by way of the Gibbs–Thomson relation [11,42]. As $T\to T_{m}^{0}$, ∆G→0 so crystallization cannot occur, $\alpha_{eq}\left(T\right)\to 0$. With increasing supercooling, ∆G grows and $\alpha_{eq}\left(T\right)$ increases, approaching a finite maximum imposed by inhibited chain movement.

The $\eta \left(T\right)$ parameter expresses how nucleation and growth kinetics constrain the extent to which the thermodynamic ceiling can be reached at a given temperature on finite timescales. Near $T_{m}^{0}$, the extent of crystallization is suppressed by the rarity of nucleation events, so the system cannot approach $∆H_{eq}\left(T\right)$ in any practically accessible time even though crystallization remains thermodynamically favorable until the equilibrium melting point is reached. Conversely, at temperatures near $T_{g}$, chain mobility is reduced, and the system is kinetically arrested despite the large thermodynamic driving force. In both limits, the maximum degree of crystallinity attainable at a given isothermal temperature, $\alpha_{max}\left(T\right)$, saturates at a fraction of the thermodynamic maximum; i.e., $\alpha_{max}\left(T\right)=\alpha_{eq}\left(T\right)\eta \left(T\right)$. In principle, in the limit of infinite time and no kinetic barriers, crystallization would approach the limit $∆H_{eq}\left(T\right)$ defined by ∆G. The kinetic asymptote is a physical limit set by nucleation and growth kinetics at each temperature, at which the residual crystallization process becomes experimentally inaccessible on any real timescale. In this work, we assume the system to have reached the kinetic asymptote at the FSC remelting curve proceeding the final interruption. We begin by expressing the equilibrium enthalpy limit as Equation (11):

(11) $$∆H_{eq}\left(T\right)\;=\;\Delta H_{f}^{o}\;\cdot \alpha_{eq}\left(T\right)$$

where $\Delta H_{f}^{o}$ is the hypothetical enthalpy of fusion of a perfectly crystalline polymer with infinitely thick lamellae (130 J/g for PEEK) [43]. According to classical equilibrium crystallization theories, $\alpha_{eq}\left(T\right)$ decreases linearly with temperature relative to the equilibrium melting point [11,44,45] and is given by Equation (12):

(12) $$\alpha_{eq}\left(T\right)\;=\;\alpha_{0}\left(1-\frac{T}{T_{m}^{0}}\right)$$

where $\alpha_{0}<1$ is the maximum equilibrium crystallinity fraction attainable at large degrees of supercooling. Mathematically, $\alpha_{eq}\in \left[0,\alpha_{0}\right]$ for $0\leq T\leq T_{m}^{0}$. The factor $\left(1-T/T_{m}^{0}\right)$ reflects the free-energy driving force for crystallization and is analogous to the degree of supercooling. The equilibrium degree of crystallinity is governed solely by the free-energy balance between melt and crystal and vanishes only at $T_{m}^{0}$. Below $T_{m}^{0}$, crystallization remains thermodynamically favorable at all temperatures, even far into the glassy regime. The observed disappearance of crystallization near and below $T_{g}$ arises from kinetic arrest, which is captured separately by the kinetic factor $\eta \left(T\right)$.

For the kinetic efficiency, we define a fractional saturation mapping by Equation (13):

(13) $$\eta \left(T\right)\;=\;\frac{\tilde{k_{iso}}\left(T\right)}{k_{c}+\tilde{k_{iso}}\left(T\right)}$$

where $\tilde{k_{iso}}\left(T\right)$ is an effective rate constant and $k_{c}>0$ is a characteristic scale parameter (Michaelis constant). The tilted notation corresponds to fit parameters within the present enthalpy model and are not to be interpreted as the canonical Hoffman–Lauritzen isokinetic rate constants in Section 3.2.1. This mapping is a normalized Michaelis–Menten-type function [46,47] originally introduced in 1913 to describe enzyme kinetics (i.e., a special case of the Michaelis–Menten expression with the saturation asymptote fixed at unity).

For $\tilde{k_{iso}}\left(T\right)$, we adopt the Hoffman–Lauritzen expression for lamellar growth, applied here in the range $T_{g}<T<T_{m}^{0}$. By itself, $\eta \left(k\right)$ is monotonic in k, but because the Hoffman–Lauritzen rate constant is unimodal with respect to temperature, $\eta \left(T\right)$ inherits the same unimodal character (i.e., it rises from near zero at both $T_{g}$ and $T_{m}^{0}$ and exhibits a maximum in between). The use of a Hoffman–Lauritzen functional form for $\eta \left(T\right)$ reflects the assumption that lamellar growth is controlled by the balance between chain mobility and thermodynamic driving force, and that this same balance determines how closely the system can approach its equilibrium crystallinity.

Based on the considerations outlined above, the final model for the maximum isothermally achievable enthalpy of crystallization is written as Equation (14):

(14) $$∆H_{max}^{\mathrm{iso}}\left(T\right)\;=\;∆H_{m}^{0}\left(1-\frac{T}{\tilde{T_{m}^{0}}}\right)\frac{\tilde{k_{iso}}\left(T\right)}{k_{c}+\tilde{k_{iso}}\left(T\right)}$$

with

(15) $$\tilde{k_{iso}}\left(T\right)=\tilde{k_{o}}exp\left(-\frac{\tilde{U^{*}}}{R\left[T-\tilde{T_{\infty }}\right]}\right)exp\left(-\frac{\tilde{K_{g}}}{T\left[\tilde{T_{m}^{0}}-T\right]f\left(T\right)}\right)$$

and

(16) $$f\left(T\right)=\frac{2T}{\tilde{T_{m}^{0}}+T}$$

where $∆H_{m}^{0}=\Delta H_{f}^{o}\alpha_{0}$ denotes the thermodynamic enthalpy ceiling at deep supercooling. The set of adjustable parameters is collected into the decision vector for minimization: $\theta_{\Delta H_{max}^{\mathrm{iso}}}=\left\{∆H_{m}^{0},k_{c},\tilde{k_{o}},\tilde{U^{*}},\tilde{K_{g}},\tilde{T_{m}^{0}},\tilde{T_{\infty }}\right\}$. The resulting fit reproduces the expected right-skewed unimodal $∆H_{max}^{\mathrm{iso}}$ profile across 155–310 °C and agrees closely with the calorimetric measurements. The optimum parameters and fit are provided in Table 5 and Figure 4d respectively.

## 4. Non-Isothermal Crystallization Kinetics Modeling

This section develops and validates a non-isothermal crystallization framework that builds on the isothermal kinetic backbone of Section 3. We suggest two closed-form scaling functions: the weight scaling factor $\omega \left(\dot{T}\right)$ and the saturation factor $\chi \left(\dot{T}\right)$. A new rate-dependent induction lag time implementation is established and finally the model is validated against a transient thermal profile representative of AM.

### 4.1. Non-Isothermal Endothermic Analysis

**Figure 5:**
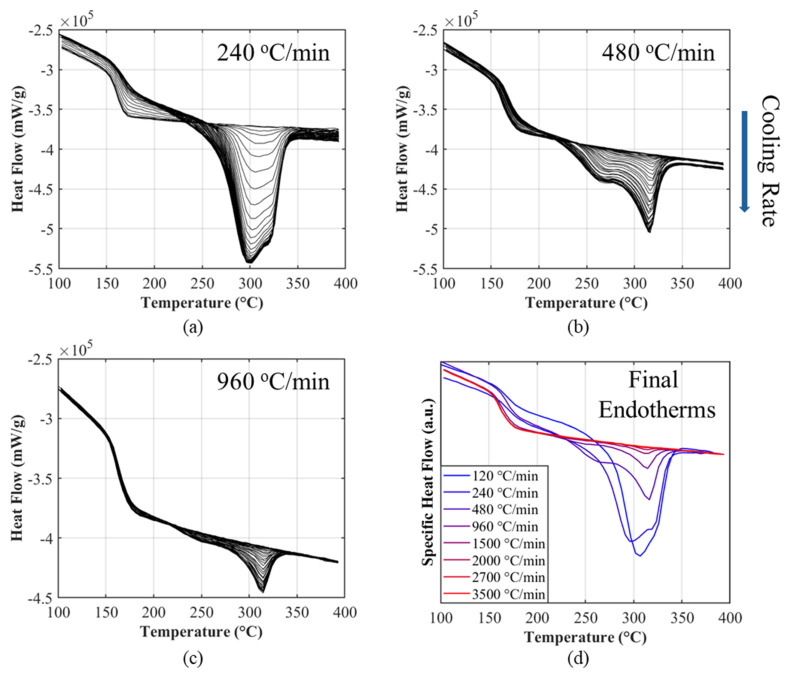
Stepwise non-isothermal melting endotherms of (**a**) 240, (**b**) 480, (**c**) 960 °C/min and (**d**) the final melting endotherms of each test.

Figure 5a–c displays the evolution of the remelting endotherms within each constant-rate test. Early interruptions exhibit a narrow, HT peak that progressively broadens toward lower temperatures as the sample is cooled further, mirroring the isothermal trend that lower crystallization temperatures yield lower LT melting peaks. Examining the final remelting endotherms in Figure 5d reveals two regimes: at (120–960 °C/min), the spectrum presents as a lopsided or bi-modal peak, whereas at (1500–2700 °C/min) only the high-temperature component persists, indicating that this population forms faster and remains observable even under strict time constraints. The enthalpy associated with each endotherm can be observed to be decreasing as temperature rate is increased. The absence (or near absence) of a remelting endotherm at the fastest rates for 2700 and 3500 °C/min is consistent with what was observed by Comelli et al.’s [24] FSC study where the author described 2700 °C/min as the critical cooling rate for PEEK with DoC below 0.7%.

### 4.2. Modified Dual-Nakamura Model

To translate the dual mechanism isokinetic parameters found in Section 3, we partition the single integral Nakamura model [20] (Equation (3)) with rate and temperature-dependent weights $w_{i}^{\mathrm{non}\text{-}\mathrm{iso}}\left(T,\dot{T}\right)$. Thus, α(T(t)) ∈ [0, 1] is written as Equation (17):

(17) $$\alpha \left(T\left(t\right)\right)=w_{1}^{\mathrm{non}\text{-}\mathrm{iso}}\left(T,\dot{T}\right)\left(1-exp\left[-{\left(\int_{0}^{t}k_{1}^{\;1/n_{1}}\;dt\right)}^{n_{1}}\right]\right)+w_{2}^{\mathrm{non}\text{-}\mathrm{iso}}\left(T,\dot{T}\right)\left(1-exp\left[-{\left(\int_{0}^{t}k_{2}^{\;1/n_{2}}\;dt\right)}^{n_{2}}\right]\right)$$

with $w_{2}^{\mathrm{non}\text{-}\mathrm{iso}}\;=\;1\;-\;w_{2}^{\mathrm{non}\text{-}\mathrm{iso}}$. The isokinetic parameters {$n_{1}$, $n_{2}$, $k_{1}\left(T\right),\;k_{2}\left(T\right)$ and $w_{1}^{\mathrm{iso}}\left(T\right)$} are those calibrated from isothermal data. Under non-isothermal conditions, the competition between the two crystallization pathways (primary and secondary) shifts with the available time at each temperature [2]. We capture this with $\omega \left(\dot{T}\right)\in [0,1]$ in Equation (18):

(18) $$w_{1}^{\mathrm{non}\text{-}\mathrm{iso}}\left(T,\dot{T}\right)\;\;=\;\;\omega \left(\dot{T}\right)\;w_{1}^{\mathrm{iso}}\left(T\right)$$

To convert α(T(t)) into $\Delta H\left(t\right)$, Equation (5) is simply restated as Equation (19):

(19) $$∆H\left(t\right)\;=\;\sum \;\alpha (T(t))\cdot \Delta H_{max}^{\mathrm{iso}}\left(T\right)$$

Previous DSC-based studies [2,5,15,20,48] did not require any further modifications for model prediction of rates between 0 and 100 °C/min. In practice, Equation (19) overpredicts ∆H(t) at faster rates. We therefore add a rate-dependent saturation factor $\chi \left(\dot{T}\right)$. The final functional equation can therefore be written as Equation (20).

(20) $$\Delta H\left(t\right)=\sum \;\alpha \left(T(t)\right)\cdot \Delta H_{max}^{\mathrm{iso}}\left(T\right)\cdot \;\chi \left(\dot{T}\right)\;$$

This is the operational non-isothermal model used in the current work. The isothermal ceiling $\Delta H_{max}^{\mathrm{iso}}\left(T\right)$ is provided by the isothermal kinetic sub-model, while the non-isothermal behavior enters only through the two rate-dependent scalers $\omega \left(\dot{T}\right)$ and $\chi \left(\dot{T}\right)$.

**Figure 6:**
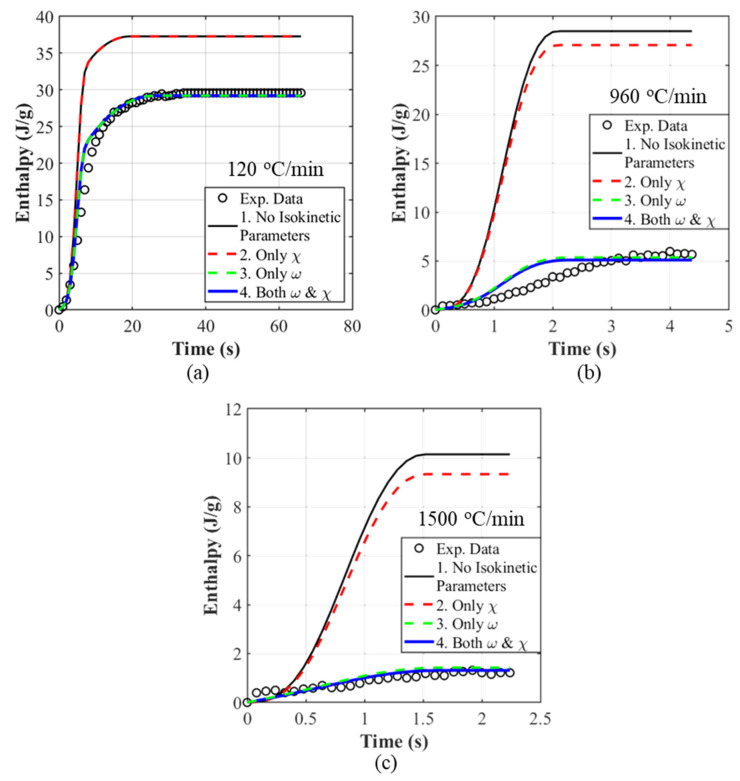
Non-isothermal model predictions using solely isokinetic parameters (red curves), and with added scalars ($\omega \left(\dot{T}\right)$ and $\chi \left(\dot{T}\right)$) (blue curves) for (**a**) 120 °C/min, (**b**) 960 °C/min, and (**c**) 1500 °C/min.

**Figure 7:**
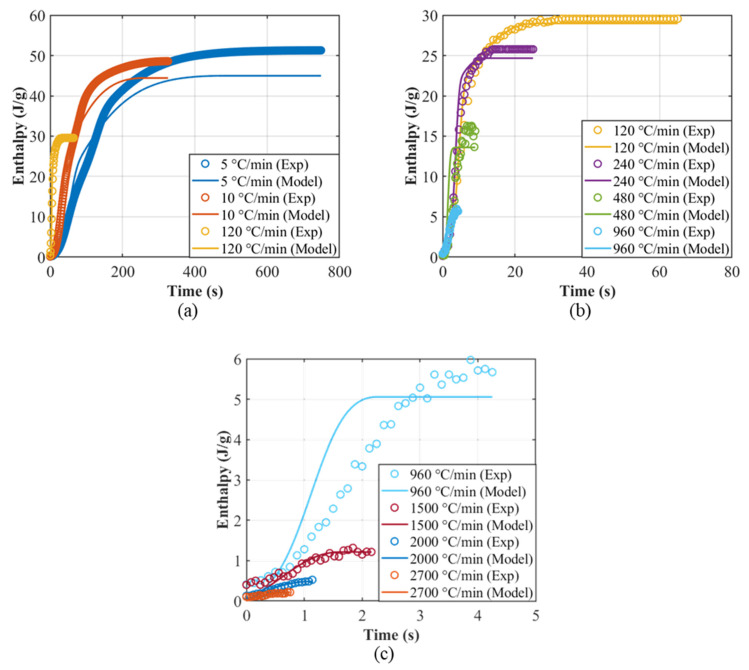
The melting enthalpy plotted against the cool-down time for (**a**) 5, 10 and 120 °C/min, (**b**) 120, 240, 480 and 960 °C/min and (**c**) 960, 1500, 2000 and 2700 °C/min.

In this work, $\omega \left(\dot{T}\right)$ and $\chi \left(\dot{T}\right)$ are calibrated directly against the constant-rate $\Delta H\left(t\right)$ evolution (Equation (20)). Both scalars reduce to unity at the isothermal limit $\left(\dot{T}\to 0\right)$, so we obtain a single, unified isothermal–non-isothermal model that preserves the calibrated isothermal kinetics while correcting the overprediction of $\Delta H\left(t\right)$ at high $\dot{T}$. The improvements to the non-isothermal model predictions with the added scaling factors can be seen in Figure 6 and all the constant-rate predictions using the calibrated model are presented in Figure 7. The $\omega \left(\dot{T}\right)$ and $\chi \left(\dot{T}\right)$ parameter calibration procedure using data obtained from non-isothermal tests is provided in the Appendix A. Figure 6 also serves as a direct comparison with the conventional dual-Nakamura implementation obtained by projecting the isothermal Velisaris–Seferis parameterization without rate-dependent corrections (red curves). The systematic overprediction at high cooling rates highlights the limitation of the classical formulation and the improvement obtained with the present rate-dependent extension.

For the final model implementation in a transient cooling profile, interpolation of the fitted scalars $\chi \left(\dot{T}\right)$ and $\omega \left(\dot{T}\right)$ is required. A monotonic decreasing trend in $\chi \left(\dot{T}\right)$ and $\omega \left(\dot{T}\right)$ is observed as a function of rate; therefore, Hill functions provided in Equations (21) and (22) were used:

(21) $$\omega \left(\dot{T}\right)\;=\;\frac{A_{0,\omega }}{1+{\left(\frac{\dot{T}}{r_{0,\omega }}\right)}^{N_{\omega }}}$$

(22) $$\chi \left(\dot{T}\right)=\frac{A_{0,\chi }}{1+{\left(\frac{\dot{T}}{r_{0,\chi }}\right)}^{N_{\chi }}}$$

The Hill function has three parameters each with the following roles: $A_{0}$ sets the low-rate plateau $\left(\omega ,\chi \to 1\;as\;\dot{T}\to 0\right)$, $r_{0}$ is a characteristic rate, and N controls the steepness. A non-linear least-squares method was used to fit the parameters subject to the following constraints:

(i) To ensure a maximum ceiling of one for scalars approaching isothermal conditions, the amplitude was set to 0.5 $\leq A_{0}\leq$1.
(ii) $r_{0}$ and N were set to strictly positive values to ensure a monotonic decreasing response with increasing $\left|\dot{T}\right|$.

**Figure 8:**
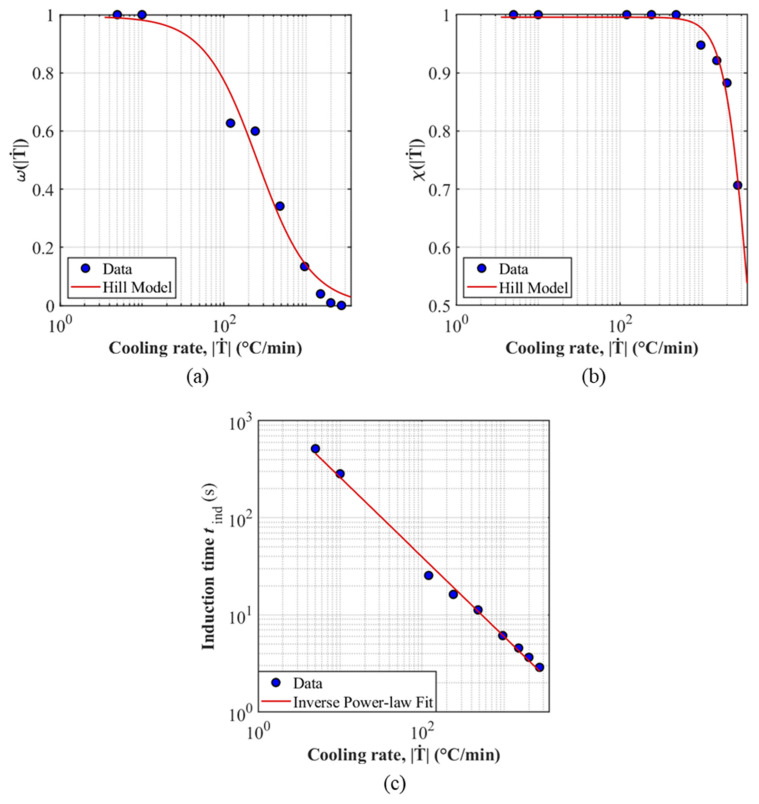
Analytical functional fits of (**a**) $\chi \left(\dot{T}\right)$, (**b**) $\omega \left(\dot{T}\right)$ and (**c**) $t_{ind,\;i}$ against non-isothermal cooling rates $\dot{T}$.

The predictions provided using these closed-form expression can be seen in Figure 8a,b and the Hill function parameters are presented in Table 6. As shown in Figure 8a, the Hill function interpolation yields a small overestimation of $\chi \left(\dot{T}\right)$ and $\omega \left(\dot{T}\right)$ at an intermediate cooling rate, which in turn gives rise to the minor local deviation observed in Figure 6.

### 4.3. Induction Time Implementation for Transient Thermal Profiles

Induction time ($t_{ind}$) is the elapsed time between imposing undercooling (or an annealing temperature for cold crystallization) and the first measurable signature of crystallization in the heat flow signal. To our knowledge, none of the Avrami-based crystallization models account for this, and its implementation represents a time shift before crystallization commences [19]. This section describes the induction time implementation in our model as a direct function of the instantaneous non-isothermal cooling rate rather than as a function of the isothermal setpoint. The choice is motivated by the intended use in processes with transient cooling profiles.

Godovsky’s [25] power law, adopted by Gordnian [15], is modified to calculate $t_{ind}$ as a function of cooling rate as seen in Equation (23):

(23) $$t_{\mathrm{ind}}\left(\dot{T}\right)\;=\;B\;{\left|\dot{T}\right|}^{-p}$$

The derivation of this inverse power law can be found in the Appendix A. The fitted parameters B and p are listed in Table 7 and the log-log transformed plot against the experimental non-isothermal induction time is provided in Figure 8c.

In all non-isothermal experiments (j=1……M), the induction period starts at T = 350 °C. Practically, the implementation of $t_{\mathrm{ind}}$ is defined as beginning at the first data point where T ≤ 350 °C; data at higher temperatures are trimmed and do not contribute to the delay. This choice mirrors the experimental protocol used to extract the non-isothermal onsets and ensures that the model-predicted $t_{\mathrm{ind}}\left(\dot{T}\right)$ is referenced to the same physical point in time.

To implement the inverse power law-based $t_{\mathrm{ind}}\left(\dot{T}\right)$, let $t_{i}$, $T_{i}$ be the sampled time and temperature below 350 °C. The local cooling rate is estimated by a backward difference calculated with Equation (24):

(24) $${\dot{T}}_{i}\;=\;\frac{T_{i}-T_{i-1}}{t_{i}-t_{i-1}}$$

At each time increment, we compute the rate-dependent induction period with Equation (25):

(25) $$t_{ind,\;i}\left(\dot{T}\right)\;=\;B\;{\left|\dot{T}\right|}_{i}^{-p}$$

and its corresponding temperature spacing

(26) $$\Delta T_{i}={\left|\dot{T}\right|}_{i}\cdot t_{ind,\;i}$$

Interpreting $t_{ind,i}$ as the local time-to-onset if the system were to continue cooling at rate ${\left|\dot{T}\right|}_{i}$, the corresponding temperature “distance-to-onset” implied at that instant is $\Delta T_{i}={\left|\dot{T}\right|}_{i}\cdot t_{ind,\;i}$. A rate-aware onset is then defined as the earliest time at which the accumulated temperature drops from $T_{1}\;=$ 350 °C equals the smallest of these distances. Formally, let $\Delta T_{min}=\min_{i}\Delta T_{i}$. The induction index $k^{*}$ is the first increment satisfying $T_{k^{*}}\leq T_{1}-\Delta T_{min}$; the operational induction time is $t_{\mathrm{ind}}=t_{k^{*}}-t_{1}$. All kinetic terms are suppressed for increments 1 to $k^{*}$, and numerical integration of the crystallization model begins at $k^{*}$. The pseudocode of the implementation can be found in Table A2 in the Appendix A.

The reason for this construction is that relying directly on $t_{ind}\left(\dot{T}\right)$ ties the decision to clock time under the most recently observed rate segment (the instantaneous rate right before crystallization proceeds ${\left|\dot{T}\right|}_{\left(k^{*}-1\right)}$), leaving it sensitive to brief decreases in rate. Converting to a temperature difference and comparing it to the accumulated drop makes the implementation aware of transient cooling profiles.

### 4.4. Model Validation

Process-level crystallization kinetics models, such as the one presented herein, are intended to predict the evolution of crystallinity under arbitrary thermal histories arising from processes like AM. Accordingly, the parameters identified in this work are not tied to a particular cooling program but are expected to remain valid for any T(t) applied to the same material system where all other process variables are held constant. The validation test presented here therefore evaluates whether the calibrated model can predict crystallization under a complex transient thermal profile representative of AM, rather than under the simple isothermal or constant-rate conditions used for parameter identification. It should be noted that material-specific factors such as molecular weight or chemical composition influence crystallization kinetics; a change in these quantities corresponds to a different material system and would require re-identification of the kinetic parameters but could be pursued by an identical framework.

To test the predictive capability of the modified dual-Nakamura framework under material processing conditions, the model (Equation (20)) was driven by the measured transient temperature–time program used in the FSC verification experiment (see end of Section 2.2). The corresponding FSC data obtained under the same programmed profile served as the experimental benchmark. No additional parameters were refitted for this test: the model used exactly the finalized parameters determined in earlier sections. Specifically, it employed the isokinetic sub-model α(t|T) with fixed $n_{1},n_{2}$ (Section 3.1); Hoffman–Lauritzen function for $k_{1}(T),k_{2}(T)$ (Section 3.2.1); branching function for $w_{1}\left(T\right),\;w_{2}\left(T\right)$ (Section 3.2.2); Michaelis–Menton-type fractional saturation function for $\Delta H_{max}^{\mathrm{iso}}\left(T\right)$ (Section 3.2.3); Hill-type dynamic scaler functions for $\omega \left(\dot{T}\right)$ and $\chi \left(\dot{T}\right)$ (Section 4.2); and the (inverse power law) rate-based $t_{\mathrm{ind}}\left(\dot{T}\right)$ (Section 4.3).

**Figure 9:**
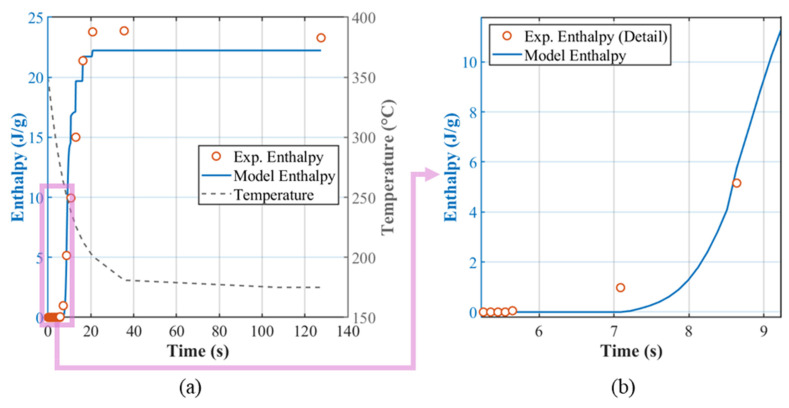
Experimental vs. model enthalpy $\Delta H\left(t\right)$ prediction for the verification test: (**a**) full response; (**b**) zoomed view of the initial stage, showing the induction time.

The recorded T(t) verification thermal profile is provided to the solver, and the output is the $\Delta H\left(t\right)$. When observing Figure 9, the first clear experimental crystallization is captured at t = 7.09 s with $\Delta H\left(t\right)$ ≈ 0.98 J/g. The rate-dependent analytical expression for $t_{\mathrm{ind}}\left(\dot{T}\right)$ with the temperature-spacing logic predicts an onset at exactly the same $t_{ind,exp}=t_{ind,model}=$ 7.09 s.

The subsequent “stair-step” features in the simulated enthalpy are not model instabilities; they reflect the abrupt cooling rate changes in the programmed thermal profile. The FSC cannot execute a continuously varying $\dot{T}$; instead, the transient program is realized as a sequence of rate plateaus. As $\omega \left(\dot{T}\right)$ and $\chi \left(\dot{T}\right)$ depend on the instantaneous cooling rate, the stepwise programming of $\dot{T}(t)$ introduces slope discontinuities in the model predictions at the programmed rate transitions. After t ≈ 35 s, the thermal profile transitions to an isothermal hold near 175 °C. By that time, the experimental sample has accumulated an enthalpy of ≈23.90 J/g. According to the model’s maximum isothermally achievable enthalpy at 175 °C (see Section 3.2.3), the ceiling at that temperature, is ≈23.30 J/g. Due to the prior transient segment already exceeding this threshold, no further crystallization is expected during the 175 °C isothermal hold. This is exactly what is observed in the FSC data and reproduced by the model. This provides an independent check on the temperature dependence encoded in the $\Delta H_{max}^{\mathrm{iso}}\left(T\right)$ relation. The coefficient of determination for this verification is *R*^2^ = 0.95.

## 5. Conclusions

This work delivers a crystallization kinetics model for PEEK that remains predictive from simple isotherms to fast, transient AM-relevant cooling profiles. Using FSC, we captured complete isothermal crystallization across the $T_{g}<T<T_{m}$ window without crystallization occurring during the ramp to the isotherm, overcoming a limitation often encountered in conventional DSC-based studies. Fitting the parallel isothermal Velisaris–Seferis model using a constrained bi-level non-linear regression revealed a mechanistic dome-shaped temperature dependence of $w_{1}\left(T\right)$ that tends to zero as T approaches $T_{g}$ and $T_{m}$. Later, a physics-based analytical expression was proposed to represent this behavior. The maximum isothermally achievable enthalpy $\Delta H_{max}^{\mathrm{iso}}\left(T\right)$ was found to vary in a right-skewed dome-shaped manner with temperature, which was modeled using a proposed physics-based temperature function.

Extending this isokinetic backbone with the integral dual-Nakamura form and two newly introduced rate-dependent scalers, $\omega \left(\dot{T}\right)$ and $\chi \left(\dot{T}\right)$, preserves the calibrated isothermal parameters while correcting systematic overpredictions of final enthalpy at high $\dot{T}$. A rate-dependent induction time implemented via a temperature difference criterion prevents sensitivity to brief rate dips in transient profiles. Collectively, these choices allow the model to predict ΔH(t) directly from the measured T(t) without recalibration. Quantitatively, this framework reproduces the ΔH(t) of an AM-relevant transient thermal profile with a high degree of accuracy (*R*^2^ = 0.95) as well as predict the exact experimental crystallization onset time (7.09 s). It also predicts the cessation of crystallization during the final 175 °C isothermal hold in the validation test by referencing the local $\Delta H_{max}^{\mathrm{iso}}\left(T\right)$.

Future work should focus on quantifying model uncertainty, clarifying the physical basis of the dual crystallization processes, and linking rate-dependent parameters to observable morphology. Statistical uncertainty analysis and repeatability studies using multiple FSC datasets would help establish confidence intervals and ensure that model parameters are robust and not overfitted to a single dataset. Further work should focus on experimentally validating the proposed mechanistic interpretation of the weight factor and the rate-dependent scalars and by correlating them with observable crystalline morphology. A combined thermal and morphological study using FSC with ex situ imaging (e.g., scanning electron microscopy or atomic force microscopy) could clarify how these model parameters are influenced by the crystal morphology. Additionally, it will be important to understand the effect of manufacturing process [49] and thermal property [50] variability on the crystallization kinetics variability.

## Figures and Tables

**Table 1:** Tabulated time vs. cooling rate $\dot{T}$.

Time t (s)	Cooling Rate $\left|\dot{T}\right|$ (°C/min)
0–0.7	1000.0
0.7–1.5	923.5
1.5–2.4	846.9
2.4–3.4	770.4
3.4–4.5	693.8
4.5–5.6	617.3
5.6–7.1	540.8
7.1–8.6	464.2
8.6–10.7	387.7
10.7–13.0	311.2
13.0–16.3	234.6
16.3–20.8	158.1
20.8–35.6	5.0
107.6–127.6	0.0

**Table 2:** Bi-level fitted parameters.

$n_{1}$	3.000		
$n_{2}$	1.001		
**Temperature (°C)**	$ln\left(k_{1}\right)$ **(s^−n1^)**	$ln\left(k_{2}\right)$ **(s^−n2^)**	$w_{1}$
170	−14.88	−5.37	0.32
180	−11.97	−4.31	0.22
200	−4.01	−3.99	0.71
220	−1.07	−5.03	0.74
245	−0.63	−4.37	0.76
260	−3.96	−3.40	0.78
290	−11.72	−4.64	0.28
300	−10.88	−5.37	0.34
310	−13.80	−5.69	0.32

**Table 3:** Optimized Hoffman–Lauritzen parameters.

Parameter $\left(\theta_{i}\right)$	*i* = 1	*i* = 2
$K_{0,i}$ (s^−2^, s^−3^)	9.16 × 10^19^	1.66 × 10^2^
$K_{g,i}$ (°K^2^)	8.03 × 10^5^	2.22 × 10^5^
$T_{\infty ,i}$ (°K)	403.23	363.15
$T_{m,i}^{0}$ (°K)	664.59	672.55
$U_{i}^{*}$ (J/mol)	3.45 × 10^3^	1.59 × 10^3^

**Table 4:** Optimized $w_{1}$ equation parameters.

$p_{1}$	0.29
$p_{2}$	0.58
λ	1.65
$p_{1}U_{1}^{*}$ (J/mol)	1.00 × 10^3^
$p_{2}U_{2}^{*}$ (J/mol)	9.24 × 10^2^
$p_{1}K_{g,1}$ (°K^−2^)	2.33 × 10^5^
$p_{2}K_{g,2}$ (°K^−2^)	1.29 × 10^5^

**Table 5:** Optimized hybrid maximum isothermally achievable crystallization enthalpy—Hoffman–Lauritzen parameters.

$∆H_{m}^{0}$ (J/g)	1931.76
$k_{c}$	8.61 × 10^23^
$\tilde{k_{o}}$ (s^−1^)	8.45 × 10^23^
$\tilde{U^{*}}$ (J/mol)	3933.47
$\tilde{K_{g}}$ (°K^2^)	5252.25
$\tilde{T_{m}^{0}}$ (°K)	683.93
$\tilde{T_{\infty }}$ (°K)	301.84

**Table 6:** Analytical fitted parameters of the scalar-valued functions of $\omega \left(\dot{T}\right)$ and $\chi \left(\dot{T}\right)$.

Scalar	$A_{0}$	$r_{0}$	n	*R* ^2^
$\omega (\dot{T})$	0.99	4.26	n: 1.33	0.98
$\chi (\dot{T})$	1.00	61.80	n: 2.96	0.98

**Table 7:** Inverse power law fitted parameters for $t_{\mathrm{ind}}\left(\dot{T}\right)$.

B	59.95
p	0.82
*R* ^2^	0.99

**Figure A1:**
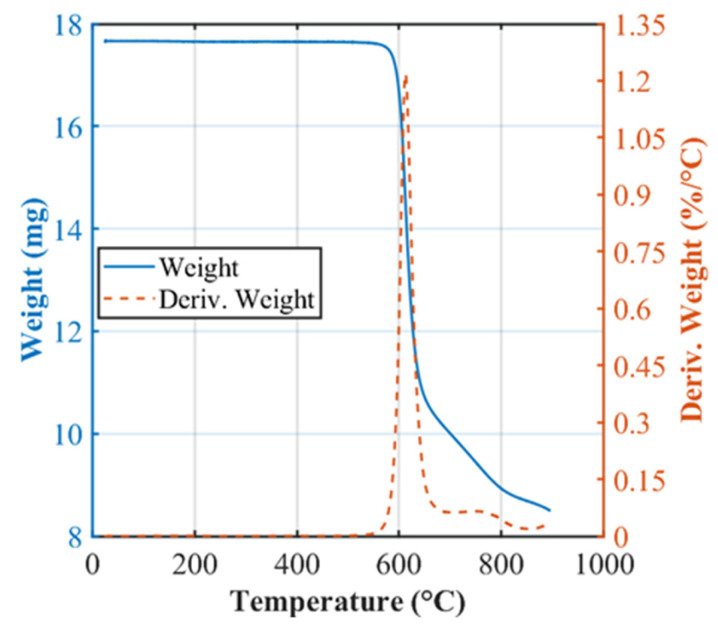
TGA performed on 17.662 mg PEEK sample at 10 °C/min from 25 °C to 900 °C.

## Data Availability

The original contributions presented in this study are included in the article. Further inquiries can be directed to the corresponding authors.

## Appendix A

### Appendix A.1. Considerations for Thermal Degradation and Thermogravimetric Analysis

Thermal degradation is a key obstacle that needed to be addressed for the successful creation, validation, and use of this model. PEEK undergoes thermal degradation when heated to temperatures in excess of 300 °C, which proceeds at exponentially increasing rates as temperature increases. Thermal degradation poses a serious issue in this work due to its enthalpy-modulating effect, which is to say that as degradation proceeds, a sample subject to repeated cycles of the same thermal profile will exhibit reduced rates of crystallization and a decrease in measurable relative enthalpy (compared to a non-degraded sample). To roughly quantify PEEK’s thermal degradation behavior, thermogravimetric analysis (TGA) was performed, with results presented in Figure A1. From this test, PEEK was shown to not undergo significant thermal degradation until ~580 °C. The data collection protocols used in this work necessitate temperatures only as high as 400 °C, meaning that the degradation captured by the TGA was not of significant concern.

However, given this model’s reliance on measured enthalpy, further calorimetric testing was undertaken to elucidate and quantify PEEK’s degradation behavior at lower temperatures and longer timespans. Schawe et al. [51] report that PEEK samples aged at 420 °C for 10 s exhibited a reduction in relative enthalpy by 20% while samples aged at 380 °C for 100 s exhibited a similar reduction. Using this baseline, aging tests were performed on the FSC at 350 °C for 5 min increments to assess relative enthalpy change. After 20 min, the relative enthalpy was shown to decrease by 20%. Moving forward for the remainder of this work, this limit was used to justify retiring FSC samples by tracking the amount of time spent above 350 °C across its tests. None of the samples used in this work spent more than 20 min above 350 °C.

### Appendix A.2. Bi-Level Non-Linear Numerical Implementation

Let the isothermal crystallization experiments spanning ≈ 155–310 °C be $T_{i},i=1$, …, M. For each temperature, the experimental data consist of a time series {$t_{i,m},\alpha_{m}^{exp}$} for m = 1,…, $N_{i}$. where $\alpha_{i,m}^{exp}$ ∈ [0, 1] is the measured relative degree of crystallinity. The exponents $n_{1}$ and $n_{2}$ are upper-level variables determined globally across all isotherms by minimizing the aggregate squared error using Equation (A1):

(A1) $${min}_{n_{1},\;n_{2}}\;=\;\sum_{i=1}^{M}{min}_{i,BL}{SSE}_{i}\left(\theta_{i,BL}|n_{1},n_{2}\right)$$

where for a given $T_{i}$, the model parameters are determined by fitting $\alpha_{i}\left(t\right)$ to the experimental data $\alpha_{i,m}^{exp}$. The (lower level) per-dataset sum of squared errors is represented by Equation (A2):

(A2) $${SSE}_{i}\left(\theta_{i,BL}|n_{1},n_{2}\right)\;=\;\sum_{i=1}^{N_{i}}{\left[\alpha_{i}\left(t_{i},m;\theta_{i,BL},n_{1},n_{2}\right)-\alpha_{i,m}^{exp}\right]}^{2}$$

Default PSO/GA settings produced $k_{1}\left(T_{i}\right),k_{2}\left(T_{i}\right)$ with implausible (T vs. k) fluctuations; increasing the swarm size (PSO) and population size (GA) to 120 and 600, respectively, yielded smooth, physically unimodal curves, consistent with the canonical bell-shaped dependence expected from growth theory [11,12].

### Appendix A.3. Hoffman–Lauritzen Parameter Estimation for $k_{i}$

The parameter $\theta_{i,k}$ estimation is posed as a non-linear least-squares problem in log space by fitting the experimentally determined $k_{i}^{exp}\left(T_{j}\right)$ across all $T_{j}$ using Equation (A3):

(A3) $$\theta_{i,k}^{opt}\;=\;\underset{\theta_{i,k}}{\min}\sum_{j=1}^{J}{\left[\ln k_{i}^{exp}\left(T_{j}\right)-\ln k_{i}^{model}\left(T_{j};\theta_{i,k}\right)\right]}^{2}$$

where

(A4) $$\ln k_{i}^{model}\left(T_{j};\theta_{i,k}\right)=\ln K_{0,i}-\frac{2U_{i}^{*}}{R\left[T-T_{\infty ,i}\right]}-\frac{2K_{g,i}}{T\left[T_{m,i}^{0}-T\right]{f\left(T\right)}_{i}}$$

The choice to formulate the objective function in logarithmic space is motivated by two considerations. First, $k_{i}^{exp}$ values span several orders of magnitude across the accessible temperature range, so working in log space prevents points at higher magnitude from dominating the fit. Second, the Hoffman–Lauritzen model is multiplicative in a transport term and a nucleation term; taking logs turns products into sums and compresses the dynamic range, which smooths and better conditions the objective, improving numerical stability.

The optimization was performed with MATLAB’s GA using a population of 400 and a maximum of 600 generations (elite fraction ≈ 2%, crossover fraction 0.8, function tolerance 1 × 10^−6^ and no parallel evaluation).

### Appendix A.4. Regression of Branching Fraction Equation for $w_{1}$

To determine the parameters of Equation (8), the model must be fitted to the available data $\left\{T_{i},w_{1}^{data}\left(T_{i}\right)\right\}$. The fitting problem is formulated as Equation (A5):

(A5) $$\underset{\theta_{w_{i}}}{\min}\sum_{j}{\left[w_{1}^{data}\left(T_{j}\right)-w_{1}^{model}\left(T;\theta_{w_{i}}\right)\right]}^{2}$$

with the decision vector $\theta_{w_{i}}=\left\{p_{1},p_{2},\lambda \right\},$ and subjected to the constraints

(A6) $$p_{1}U_{1}^{*}>p_{2}U_{2}^{*},\;p_{1}K_{g,1}>p_{2}K_{g,2},\;p_{1}>0,\;p_{2}>0,\lambda >0$$

MATLAB’s genetic algorithm (GA) was used to minimize the least-squares error between $w_{1}^{data}(T)$ and $w_{1}^{model}(T)$.

### Appendix A.5. Regression of Fractional Saturation Mapping for $\Delta H_{max}^{\mathrm{iso}}$

For this non-linear regression, the set of adjustable parameters is collected into the decision vector $\theta_{\Delta H_{max}^{\mathrm{iso}}}=\left\{∆H_{m}^{0},k_{c},\tilde{k_{o}},\tilde{U^{*}},\tilde{K_{g}},\tilde{T_{m}^{0}},\tilde{T_{\infty }}\right\}$.

These parameters are obtained by minimizing the least-squares error between the experimental measurements $\Delta H_{max,exp}^{\mathrm{iso}}\left(T_{i}\right)$ and the model predictions $\Delta H_{max}^{\mathrm{iso}}\left(T_{i};\theta \right)$ using Equation (A7):

(A7) $$\theta_{\Delta H_{max}^{\mathrm{iso}}}^{opt}\;=\;\underset{\theta_{i,\Delta H_{\max}^{\mathrm{iso}}}}{\min}\sum_{i=1}^{N}{\left[\Delta H_{max,exp}^{\mathrm{iso}}\left(T_{i}\right)-\Delta H_{max,model}^{\mathrm{iso}}\left(T_{i};\theta_{\Delta H_{max}^{\mathrm{iso}}}\right)\right]}^{2}$$

Parameter estimation for Equations (14)–(16) was performed in MATLAB using GA. The resulting fit reproduces the expected right-skewed unimodal $∆H_{max}^{\mathrm{iso}}$ profile across 155–310 °C and agrees closely with the calorimetric measurements.

### Appendix A.6. Regression of Rate-Dependent Scaling Factors $\omega (\dot{T})$ and $\chi (\dot{T})$

To maintain physical relevance, bound constraints are imposed for every run: 0 ≤ $\omega_{j}$ ≤ 1 and 0 < $\chi_{j}$ ≤ 1. The constrained optimization led to a smooth predictable trend of $\omega_{j}$ and $\chi_{j}$ against $\dot{T}$. The fitted $\omega_{j}$ and $\chi_{j}$ values for each rate are provided in Table A1.

Consider M non-isothermal runs indexed by j = 1,…,M. Each run provides trimmed cumulative–enthalpy data pairs ${\left\{t_{j,m},\;y_{j,m}^{exp}\right\}}_{m\;=\;1}^{N_{j}}$ (units J/g). For any parameter pair ($\omega_{j}$, $\chi_{j}$), the model returns a predicted cumulative curve as denoted by Equation (A8):

(A8) $$y_{j}^{\mathrm{model}}\left(t;\omega_{j},\chi_{j}\right)\;=\;\chi_{j}\;G_{j}\left(t;\omega_{j}\right)$$

where $G_{j}\left(t;\omega_{j}\right)$ is generated by the isothermal kinetic model parameters discussed in the previous chapter (pre-specified $k_{1}(T)$, $k_{2}(T)$, $n_{1}$, $n_{2}$, $w_{1}\left(T\right)$, and $\Delta H_{max}^{\mathrm{iso}}\left(T\right)$). During this stage, only the two scale factors vary; all other isokinetic parameters with their functional forms remain fixed. The unknowns across all datasets are stacked into a single vector $\theta_{\omega ,\chi }$ $=\;(\omega_{1},\chi_{1},\omega_{2},\chi_{2},\ldots ,\omega_{M},\omega_{M})$. This layout allows a single simultaneous optimization over all runs.

Parameters are obtained by minimizing the total sum of squared residuals across all runs as given in Equation (A9):

(A9) $$J\left(\theta_{\omega ,\chi }\right)\;=\;\sum_{j=1}^{M}\sum_{m=1}^{N_{j}}{\left[\;y_{j}^{\mathrm{model}}\left(t_{j,m};\omega_{j},\chi_{j}\right)-y_{j,m}^{exp}\right]}^{2}$$

Both $\omega_{j}$ and $\chi_{j}$ are scalers: $\chi_{j}$ multiplies the entire equation directly, and $\omega_{j}$ scales the mixture weight inside $G_{j}$. As both knobs affect overall amplitude, the pair ($\omega_{j}$, $\chi_{j}$) is coupled. An unconstrained pass where $\omega_{j}$ and $\chi_{j}$ were allowed to freely vary showed no consistent trend with cooling rate and traded off against each other. To keep solutions physical, bound constraints are imposed for every run: 0 ≤ $\omega_{j}$ ≤ 1 and 0 < $\chi_{j}$ ≤ 1.

### Appendix A.7. Derivation of Rate-Dependent Induction Time

Godovsky’s [25] power law, adopted by Gordnian [15], models the decay of induction time with increasing undercooling. It is expressed as Equation (A10):

(A10) $$t_{ind}\left(T\right)\;=\;t_{m}{\left(T_{m}^{0}-T\right)}^{-c}\;=\;t_{m}\Delta T^{-c}$$

where $t_{m}>$0 and c>0 are the empirical fitting parameters, and $\Delta T=T_{m}^{0}-T$. The additivity assumption says that, over a small time slice dt spent at $T\left(t\right)$, we accumulate a fraction $dt/t_{ind,i}\left(T\left(t\right)\right)$ of the work needed to trigger the first detectable enthalpy. Induction period ends when the accumulated fraction reaches unity [15,23]:

(A11) $$\int_{0}^{t_{ind}}\frac{dt}{t_{ind,i}\left(T\left(t\right)\right)}\;=\;1$$

Substituting in Equation (A10) leads to

(A12) $$\frac{1}{t_{m}}\int_{0}^{t_{ind}}{\left[\Delta T\left(t\right)\right]}^{c}\;dt=1$$

If the rate is (locally) constant, $T\left(t\right)=T_{m}^{0}-\left|\dot{T}\right|\;t$, then $\Delta T\left(t\right)=\left|\dot{T}\right|\;t$. Substituting in Equation (A12) gives

(A13) $$\frac{1}{t_{m}}\int_{0}^{t_{ind}}{\left(\left|\dot{T}\right|\;t\right)}^{c}\;dt=\frac{{\left|\dot{T}\right|}^{c}}{t_{m}}\cdot \frac{t_{\mathrm{ind}}^{\;c+1}}{c+1}=1$$

Simplifying to Equation (A14):

(A14) $$t_{ind}\;=\;{\left[\left(c+1\right)t_{m}\right]}^{\frac{1}{c+1}}\cdot {\left|\dot{T}\right|}^{-\frac{c}{c+1}}$$

This is exactly an inverse power law as a function of cooling rate:

(A15) $$t_{\mathrm{ind}}\left(\dot{T}\right)\;=\;B\;{\left|\dot{T}\right|}^{-p}$$

with

(A16) $$B={\left[\left(c+1\right)\;t_{m}\right]}^{\frac{1}{c+1}}$$

and

(A17) $$p=\frac{c}{c+1}\in \left(0,1\right)$$

**Table A1:** The fitted dynamic scaling factors for each run together with the per-run R^2^ values for fit quality.

Cooling Rate $\dot{\left|T\right|}$ (°C·min^−1^)	$\omega_{j}$ (Fitted)	$\chi_{j}$ (Fitted)	R^2^_j_
5	1.00	1.00	0.88
10	1.00	1.00	0.94
120	0.63	1.00	0.97
240	0.60	1.00	0.95
480	0.34	1.00	0.51
960	0.13	0.95	0.71
1500	0.04	0.92	0.76
2000	0.01	0.88	0.81
2700	0.00	0.71	0.17

**Table A2:** Pseudocode for the dynamic cooling rate induction time logic.

**Pseudocode**
- Set $t_{1}=\mathrm{first}\{t(T\leq 350{}^{\circ}C)\}$; - Rebase time to $t-t_{1}$; - Compute $r_{i}=\left|{\dot{T}}_{i}\right|$ °C/s by finite differences; - Evaluate $t_{ind,i}\left(\dot{T}\right)=B\;r_{i}^{-p}$ and $\Delta T_{i}=r_{i}\cdot t_{ind,i}$; - $\Delta T_{min}=\min_{i}\Delta T_{i}$; - Find $k^{*}$ where $T_{k^{*}}\leq 350{}^{\circ}C-\Delta T_{min}$; - Set $t_{ind}=t_{k^{*}}-t_{1}$; - Suppress all kinetic integrands for increments 1: $k^{*}$ and begin integration at $k^{*}+1$.
